# Psoralen-mediated regulation of osteogenic differentiation of periodontal ligament stem cells: involvement of the mTOR pathway

**DOI:** 10.3389/fcell.2025.1634945

**Published:** 2025-07-11

**Authors:** Yujia Wang, Hongbo Zhang, Jie Yu, Jin Jing, Zhaojiang Fu, Yuanping Hao, Qihang Huang, Ruibin Ma, Yingjie Xu, Yingtao Wu

**Affiliations:** ^1^ Qingdao Stomatological Hospital Affiliated to Qingdao University, Qingdao, Shandong, China; ^2^ Zibo Stomatological Hospital, Zibo, China; ^3^ School of Stomatology, Shandong Second Medical University, Weifang, China

**Keywords:** PDLSCs, Psoralen, osteogenic differentiation, mTOR, periodontitis

## Abstract

**Background:**

Chronic periodontitis is a prevalent inflammatory and destructive oral disease, and its primary treatment is to control the development of inflammation and promote the regeneration of periodontal tissue. Psoralen (Pso) has been shown to promote the osteogenic differentiation of periodontal ligament stem cells (PDLSCs), suggesting its potential as a therapeutic agent for osteogenic regeneration.

**Methods:**

Network pharmacology and transcriptomic sequencing were exploited to screen target genes of Pso in PDLSCs, lentiviruses were employed to interfere with the target gene, and RT-qPCR was conducted to assess the expression levels of osteogenesis-related factors. Pso-loaded mesoporous polydopamine (MPDA-Pso) nanoparticles were constructed and evaluated *in vitro*, and *in vivo* osteogenesis was assessed in rats with alveolar bone defects.

**Results:**

Network pharmacology analysis revealed that the mammalian target of rapamycin (mTOR) was a potential target of Pso, and Pso significantly modulated the expression levels of mTOR in PDLSCs and markedly enhanced osteogenic differentiation. However, Pso did not significantly alter osteogenesis-related genes in PDLSCs after mTOR-inhibitor treatment. We also confirmed that MPDA-Pso nanoparticles promoted the expression of osteogenesis-related genes in PDLSCs; and compared with the control group, observed that the mass of new bone was augmented in the MPDA-Pso group.

**Conclusion:**

Pso was shown to promote the osteogenic differentiation of PDLSCs, and we postulate that this differentiation was facilitated in the LPS-induced inflammatory microenvironment via inhibition of the autophagy-related mTOR-signaling pathway. Additionally, the MPDA-Pso nanoparticles we developed promoted osteogenesis.

## 1 Introduction

Periodontitis is a chronic, progressive inflammatory disease that affects periodontal tissue, and its primary clinical manifestations include the formation of periodontal pockets and varying degrees of alveolar bone resorption. In advanced cases, periodontitis can lead to tooth loosening, displacement, or even total loss (Kinane, 2000; Sanz et al., 2020a; Teles et al., 2022). As the predominant cause of tooth loss in adults, significantly impacting patient quality of life. Epidemiologic data reveal its worldwide prevalence, affecting approximately 11% of the population in its severe form, ranking as the sixth most prevalent disease worldwide (Kinane, 2000; Kwon et al., 2021). The primary goal of periodontitis treatment is to control inflammation, prevent further destruction of periodontal tissue, and promote regeneration. Primary treatments such as supragingival scaling and root planning are considered the gold standard for chronic periodontitis (Sanz et al., 2012; Herrera et al., 2022; Sanz et al., 2020b). However, periodontitis is also closely related to various systemic diseases such as diabetes and chronic kidney disease. To effectively manage inflammation and prevent infections resulting from systemic factors, adjunctive treatment with antibiotics is often necessary (Graziani et al., 2000; Liccardo et al., 2019). Nonetheless, antibiotics can lead to drug resistance and other side effects, so it is urgent to uncover new drugs for periodontal local treatment that manifest anti-inflammatory and antibacterial properties while minimizing side effects in order to facilitate the regeneration of periodontal bone tissue.

Psoralen (Pso), a linear furanocoumarin compound, is the principal bioactive constituen of the traditional Chinese herb *Psoralea corylifolia L.*, and is a member of the linear furanocoumarin family. Pso is recognized for its diverse biologic activities, including anti-inflammatory, antibacterial, anti-tumor, and estrogen-like effects—specifically facilitating the promotion of osteogenic differentiation (Li et al., 2018; Jamalis et al., 2020); and has been applied widely to the clinical arena. Researchers indicate that Pso enhances the proliferation and vitality of bone marrow mesenchymal stem cells while also promoting their osteogenic differentiation (Huang et al., 2021; Yuan et al., 2016). We previously revealed that Pso elevated levels of the anti-inflammatory factor IL-10 in serum and reduced the secretion of the pro-inflammatory factor TNF-α (Liu et al., 2021). However, the precise mechanism by which Pso promotes osteogenic differentiation of periodontal ligament stem cells (PDLSCs) remains unclear.

Autophagy functions as a self-protective mechanism that cells use to against oxidative stress, hypoxia, and exogenous microorganisms. It facilitates the removal of harmful substances and factors generated during alveolar bone resorption, and occupies a crucial role in regulating the differentiation and functions of cells involved in bone metabolism (Guo et al., 2021; Wang et al., 2023; Liu et al., 2015). Autophagy also facilitates cellular metabolism and assists in maintaining homeostasis within the intracellular environment. Studies have revealed that autophagy is essential for the proliferation and osteogenic differentiation of bone marrow mesenchymal stem cells (BMSCs), with the mammalian target of rapamycin (mTOR)-signaling pathway significantly contributing to this process (Nuschke et al., 2014). Network pharmacology studies have also identified mTOR as a potential target for Pso. We therefore speculate that Pso mediates the osteogenic regulatory effect through the mTOR signal-transduction pathway.

Pso, however, exhibits limited solubility in water, rendering it unsuitable for topical periodontal administration. Consequently, optimizing its delivery methods and surface properties are challenges that must be addressed prior to clinical application. Mesoporous polydopamine (MPDA) has been demonstrated to serve as a drug carrier that manifests favorable mechanical compatibility, with the aromatic rings on the surface of the polydopamine facilitating drug loading via hydrophobic-hydrophobic interactions (Jiang et al., 2022; Hu et al., 2022). Furthermore, the substantial surface area of the pores enhances drug-loading capacity and encapsulation efficiency, providing a foundation for effective drug delivery and targeted release (Zhu et al., 2021; Ma et al., 2022; Ch et al., 2016); this then enables the precise localization for periodontal drug therapy. Additionally, MPDA demonstrates excellent water solubility, significantly improving the water dispersibility of hydrophobic drugs post-loading. These physicochemical properties have therefore recently led to the extensive exploration of MPDA as a drug carrier.

Thus, to improve the properties of Pso, we encapsulated hydrophobic Pso within MPDA to create novel MPDA-Pso nanoparticles, and incorporated them into PDLSCs *in vitro* and alveolar bone defects in rats *in vivo*. We hypothesized that Pso would promote osteogenic differentiation *in vivo and in vitro*, and that it would reflect a potential for development into a novel drug for clinical application.

## 2 Materials and methods

### 2.1 Effect of pso on osteogenic differentiation of PDLSCs

#### 2.1.1 Culture of PDLSCs

PDLSCs were isolated from the periodontal membrane of healthy first premolars reserved for extraction due to orthodontic needs of patients with no prior history of periodontal disease. The subjects, aged between 18 and 25 years, received treatment at the Oral and Maxillofacial Surgery Department of Qingdao Stomatological Hospital and showed no underlying systemic conditions. Primary PDLSCs were cultured in medium supplemented with 20% fetal bovine serum (FBS, Pricella, China), and all other cultures were maintained with a 10% concentration. This study was approved by the Ethics Committee of Qingdao University (QDU-HEC-2024266).

#### 2.1.2 Adipogenic differentiation

When PDLSCs reached their third passage, they were plated at a density of 1 × 10^5^ cells per well in a six-well plate and subjected to adipogenic differentiation using specific medium (Pricella, China), with medium refreshed every 3 days for 21 days. Following this period, the cells were fixed in 4% paraformaldehyde (Solarbio, China), rinsed with PBS, and stained with oil red O (Pricella, China) for subsequent microscopic examination.

#### 2.1.3 Osteogenic differentiation

After the third passage, PDLSCs were similarly plated at a density of 1 × 10^5^ cells per well in a six-well plate and induced with osteogenic differentiation medium (Pricella, China). The cells were then fixed with 4% paraformaldehyde, rinsed with PBS, and subjected to alizarin red (Pricella, China) and alkaline phosphatase (ALP) staining (Pricella, China) for microscopic examination.

#### 2.1.4 Flow-cytometric identification

The cell density at the third-generation of PDLSCs was adjusted to 1 × 10^6^ cells/mL. The cellular suspension was aliquoted into centrifuge tubes at 100 µL per tube, and antibodies generated against CD34, CD45, CD73, CD90, and CD105 (Pricella, China) (at 1:50 dilutions) were added separately, while the control group received an equivalent volume of PBS. All cells were then incubated at room temperature in the dark for 20 min. After centrifugation, the supernatant was discarded, and the pellet was washed twice with PBS. The cells were resuspended in 500 µL of PBS for analysis on a flow cytometer (CytoFLEX; Beckman Coulter, Fullerton, California, United States).

#### 2.1.5 CCK-8 assay

PDLSCs were seeded at a density of 2 × 10^4^ cells per well in a 96-well plate and cultured with a gradient of lipopolysaccharide (LPS) concentrations at 0 μg/mL, 20 ng/mL, 50 ng/mL, 100 ng/mL, 150 ng/mL,and 200 ng/mL to screen the concentration of LPS in the simulated periodontitis microenvironment. On this basis, the effective concentration of Pso was screened at concentrations of 2 μg/mL, 5 μg/mL, 10 μg/mL, 15 μg/mL, 20 μg/mL, and 25 μg/mL. We evaluated cellular proliferation using a microplate reader (Bio-Tek, United States).

#### 2.1.6 Pso promotes osteogenesis of PDLSCs

PDLSCs were co-cultured with Pso after LPS-induction and the LPS-induced stem cells served as the control group. We followed the same experimental protocol as in section 2.1.2, with alizarin red staining and ALP staining performed.

### 2.2 Network construction of the drug-target pathway

#### 2.2.1 Network pharmacology screening of drug targets

Network pharmacology analysis was employed to screen the potential targets of Pso in relation to chronic periodontitis (Beijing Allwegene Tech.). We implemented this approach to determine the specific targets that Pso may affect with respect to chronic periodontitis. Pathways with a significant enrichment of target genes were then analyzed to construct a drug-target-pathway network.

#### 2.2.2 Transcriptomics

Cells were allocated to four groups: PDLSCs, PDLSCs induced by LPS, PDLSCs cultured with Pso, and cells cultured with both LPS/PSO. Total RNA was extracted using TRIzol reagent (Takara, Japan), and high-throughput sequencing was conducted by Beijing Allwegene Tech., with *P* < 0.05 designating differentially expressed genes (DEGs). We performed gene Ontology (GO) and Kyoto Encyclopedia of Genes and Genomes (KEGG) enrichment analyses to identify the pathways associated with the DEGs.

#### 2.2.3 Detection of osteogenesis-related targeted protein

Total cell protein from all groups noted above were extracted using RIPA lysis solution (Elabscience, China) on ice, and a BCA kit (Elabscience, China) was employed to determine protein concentration. With GADPH as the internal reference, ALP (1: 1000; (Abcam, United Kingdom), OCN (1: 5000; Abcam, United Kingdom), Runt-related transcription factor 2 (RUNX2; 1: 1000; Abcam, United Kingdom), and mTOR (1: 1000; Boster, United States) were added sequentially and proteins incubated at 4°C overnight. After incubation with second antibody, ECL luminescent reagent (Elabscience, China) was added to observe HRP-labeled protein bands.

#### 2.2.4 Detection of mTOR pathway-related genes

Total RNA from all groups noted above was extracted using TRIzol reagent, and the expression levels of PI3K, LC3, Deptor, PI3K, and WIPI 1 (Sangon Biotech, China) were determined by real-time q-PCR. GADPH (Sangon Biotech, China) was used as the internal reference, and relative expression levels were calculated using the 2^−ΔΔCT^ method. All analyses were repeated three times. The primers that we used were PI3K, 101 CTT TGC GAC AAG ACT GCC GAG AG (forward) and 101 CGC CTG AAG CTG AGC AAC ATC C (reverse); WIPI 1, 114 TGC TTG GCT CAG GAA CAA CAG AAG (forward) and 114 GCA CCG TGG AGG CTG AAG ATG (reverse); LC3, 89 TCT GAG GGC GAG AAG ATC CGA AAG (forward) and 89 TCC AGG TCT CCT ATC CGA GCT TTG (reverse); RagC, 139 CAG CGG CAA GTC CTC CAT CC (forward) and 139 CAT GTA GTC ATC CTG TGC GTC AAT G (reverse); and Deptor, 118 TTG TGG TGC GAG GAA GTA AGC C (forward) and 118 AGG ACA TTG AGC CCG TTG ACA G (reverse).

### 2.3 The role of the mTOR-signaling axis in osteogenic differentiation

#### 2.3.1 mTOR inhibitors

Rapamycin is a potent and specific mTOR inhibitor and an autophagic activator (Rangaraju et al., 2010; Edwards and Wandless, 2007), and can be used to effectively inhibit the expression of mTOR. PDLSCs were cultured with rapamycin (MCE, United States) and dissolved in DMSO (Solarbio, China), and PCR was conducted to verify mTOR gene expression.

#### 2.3.2 Detection of osteogenesis-related factors

We added Pso to the control group to form the experimental group, extracted RNA, and implemented PCR to evaluate the changes in gene-expression levels. Cells were then allocated to the control group or mTOR-inhibitor group. The gene and protein expression levels for Rag C and for the osteogenic genes ALP and RUNX2 were ultimately ascertained.

#### 2.3.3 Lentiviral transfection of PDLSCs

Lentiviruses for Rag C knockdown were constructed by Jikai Gene (Shanghai, China), and PDLSCs in optimal growth conditions were selected for lentiviral transfection. After 16 h of PDLSC transfection with lentivirus, normal medium was replaced, cultures were continued for an additional 72 h, and cells were observed under a fluorescence microscope. Following successful transfection, puromycin was employed to select for cell lines with stable expression, and PCR was utilized to assess expression changes in ALP and RUNX2 genes.

### 2.4 Preparation and characterization of MPDA-Pso nanoparticles

#### 2.4.1 Synthesis of MPDA nanoparticles

After mixing absolute ethanol and purified water, F127 and TMB were added and the mixture stirred for 30 min at room temperature. Dopamine hydrochloride (Aladdin, China) and Tris were then added, and the mixture was stirred for 24 h at room temperature. The mixture was centrifuged at 12,000 rpm for 10 min, and MPDA was obtained by washing the mixture of absolute ethanol/acetone (v/v = 2:1) three times by centrifugal precipitation.

#### 2.4.2 Preparation of MPDA-Pso composites

MPDA and Pso were dispersed in absolute ethanol by ultrasonic shock. The evenly distributed MPDA and Pso were subsequently mixed and stirred in a magnetic stirrer for 24 h at room temperature.

#### 2.4.3 Characterization of MPDA and MPDA-Pso

The surface morphology and diameter of MPDA and MPDA-Pso composites were determined by transmission electron microscopy (TEM). The zeta potential was used to assess the stability of MPDA and MPDA-Pso, and the peaks for MPDA and MPDA-Pso were characterized using a Fourier transform infrared spectrometer (NICOLET iS50 FT-IR) and ultraviolet spectrophotometer.

### 2.5 Effect of MPDA-Pso nanoparticles on osteogenic differentiation

#### 2.5.1 Alizarin red staining

PDLSCs were added to six-well plates at 2 × 10^5^ cells per well and divided into four groups: an MPDA group, Pso group, MPDA-Pso group, and blank control group. The medium was changed every 3 days for 21 days, and the cells were then fixed with 4% paraformaldehyde, washed with PBS, and stained with alizarin red.

#### 2.5.2 Alkaline phosphatase staining

PDLSCs were added to six-well plates at a concentration of 2 × 10^5^ cells per well and grouped as 2.5.1 cells, and we changed the solution every 3 days and co-cultured them for 14 days. The cells were then fixed in 4% paraformaldehyde (Solarbio, China) for 30 min, washed with PBS, and stained with an ALP staining kit.

#### 2.5.3 Quantification of alkaline phosphatase

After 14 days, the existing medium was removed, and the cells were fixed with 4% paraformaldehyde and rinsed with PBS. The cells in each group were then lysed on ice. Protein concentration was assessed with a BCA kit, and absorbance was measured at 520 nm after treatment with an ALP kit, and ALP activity was calculated according to the formula.

#### 2.5.4 Detection of osteogenesis-related gene expression in PDLSCs

PDLSCs were seeded in six-well plates and grouped as in 2.5.1 above. After 7 days of culture, the RNA was extracted and its concentration was analyzed. The expression of ALP was determined by q-PCR using QuantiNova PCR Kits (QIAGEN, Germany). We adopted GAPDH as an internal reference for all samples, and the results were quantified as relative expression using the 2^−ΔΔ^Ct method. The primers that we used for ALP were 92 ACT CTC CGA GAT GGT GGTGGT G (forward) and 92 CGT GGT CAA TTC TGC CTC CTT CC (reverse). Three replicates of the experiment were performed for each group.

#### 2.5.5 Effects of MPDA-Pso nanoparticles on osteogenesis in rats with alveolar bone defects

To verify the osteogenic effects of MPDA-Pso nanoparticles *in vivo*, we anesthetized eight-week-old Sprague-Dawley rats using sodium pentobarbital, extracted their maxillary first molar, and established an alveolar bone-defect model. Following tooth extraction we applied a gelatin sponge, gelatin sponge combined with MPDA, gelatin sponge combined with MPDA-Pso, or a control group with no material to the alveolar socket. The maxilla (which included the alveolar bone-defect site) was dissected from the sacrificed rats on days seven and 28. Micro-computed tomography (micro-CT) was employed to reconstruct 3D images of alveolar bone defects and to conduct a quantitative analysis. We also performed hematoxylin and eosin (H&E) staining to evaluate the degree of alveolar bone healing. Our experimental procedures complied with the NIH Guide for the Care and Use of Laboratory Animals (United States); and the present study was approved by the Ethics Committee of Qingdao University (QDU-AEC-2024475).

## 3 Results

### 3.1 Effect of pso on osteogenic differentiation of PDLSCs

#### 3.1.1 Culture and identification of PDLSCs

PDLSCs have the potential of self-renewal and multi-lineage differentiation, and are vital to the repair of periodontal tissue damage and the regenerative treatment of periodontal disease (Calabrese, 2021). These cells are also the primary source of newly attaching cells following periodontitis treatment (Yu et al., 2017). We first cultured PDLSCs and identified them, and observed that the primary stem cells grew radially around the tissue block and were arranged in a vortex shape, exhibiting a typically long spindle shape (S1 A). After osteogenic and adipogenic induction, mineralized red nodules (S1 C) and pink lipid droplets (S1 D) were observed microscopically. Flow-cytometric analysis showed that the primary stem cells exhibited characteristics of mesenchymal stem cells (MSCS)—i.e., the cells were positive for CD73, CD90, and CD105 (S1 F), while they were negative for CD34 and CD45 (S1 E). These results indicated that the cells we extracted were, in fact, PDLSCs, and that PDLSCs manifested the basic characteristics of BMSCs.

#### 3.1.2 Effects of Pso on PDLSCs

**FIGURE 1:**
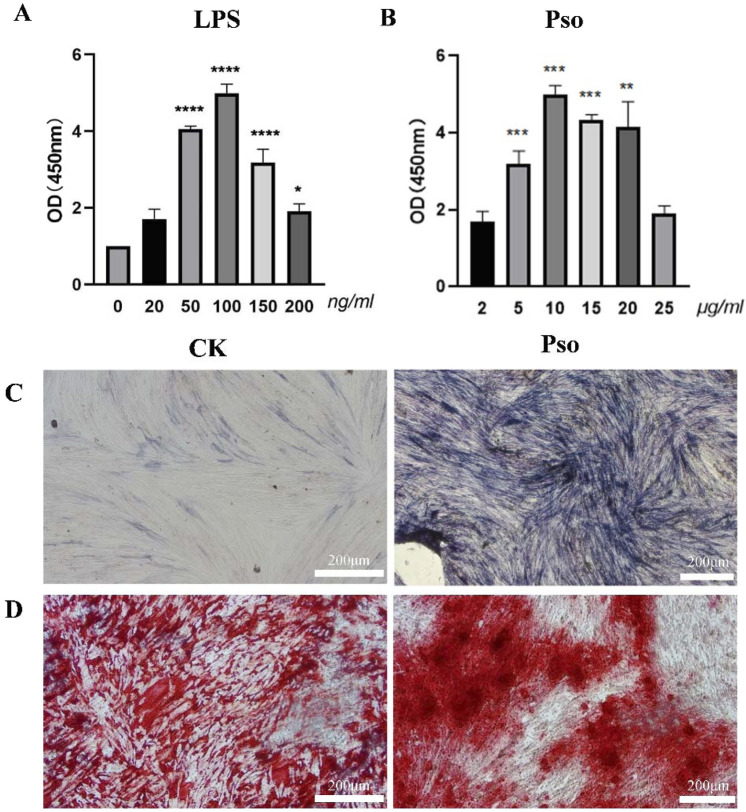
Effects of Pso on PDLSCs. **(A)** Impact of various concentrations of Pso on the viability of PDLSCs. **(B)** Influence of different concentrations of LPS on the viability of PDLSCs. **(C)** Representative images of ALP staining. CK, PDLSCs only; Pso, PDLSCs plus Pso. **(D)** Representative images of alizarin red staining (scale bar = 200 μm). Data are expressed as means ± SD. *P < 0.05, **P < 0.01, ***P < 0.001, and ****P < 0.0001.

LPS is known to stimulate the production of pro-inflammatory cytokines *in vitro*, and has been employed to simulate periodontal inflammatory disease (Stemmler et al., 2021). In order to emulate the periodontal microenvironment, PDLSCs were co-cultured with LPS, according to the references and our CCK-8 assay showed that 100 ng/mL LPS can simulate periodontitis environment and did not inhibit the proliferation of PDLSCs (Figure 1A). On this basis, we found that 10 μg/mL Pso significantly promoted the activity of PDLSCs (Figure 1B). Additionally,We noted that as LPS concentration increased, the enhancement in cellular vitality became less pronounced. Consequently, we selected 10 μg/mL Pso and 100 ng/mL LPS for subsequent experiments. ALP staining revealed a distinct blue-black coloration in the Pso group relative to the control group (Figure 1C), denoting a higher production of ALP in the former group. Additionally, alizarin red staining demonstrated an increased presence of calcium nodules in the Pso group (Figure 1D), further supporting the conclusion that Pso promoted the osteogenic differentiation of PDLSCs.

### 3.2 Drug-target-pathway network construction

#### 3.2.1 Network pharmacology screening of drug targets

**FIGURE 2:**
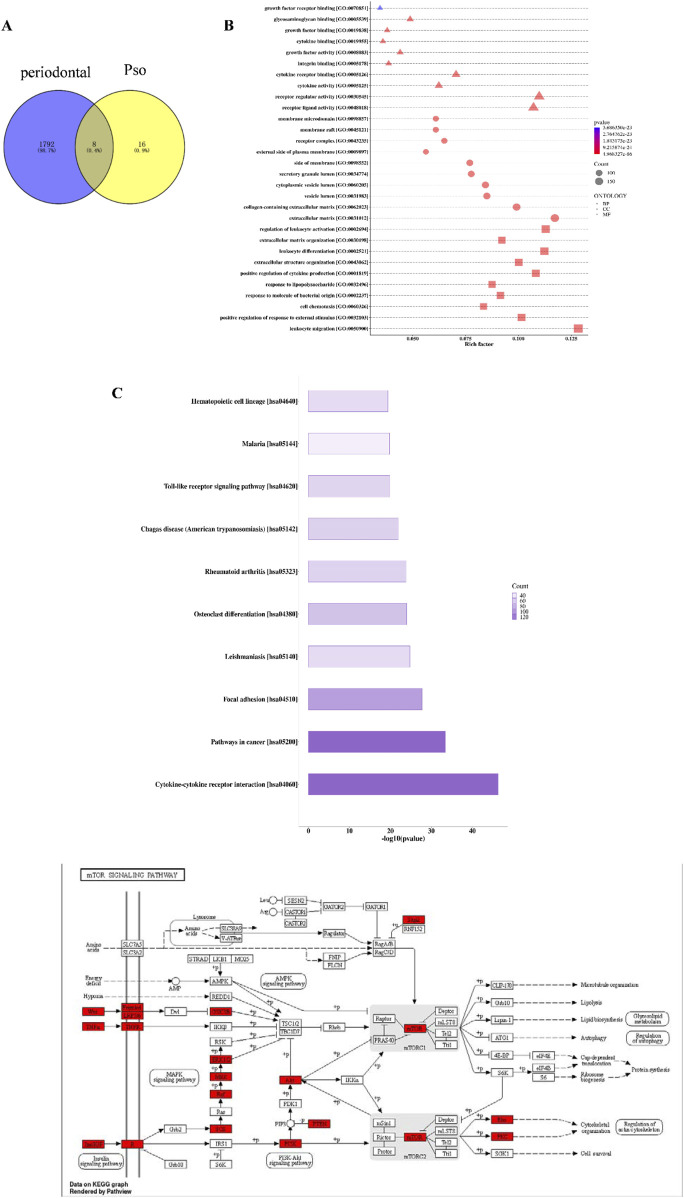
Network pharmacology results. **(A)** Venn diagram of Pso and chronic periodontitis targets. **(B)** Bubble chart of GO-enrichment analysis for Pso targets. The horizontal axis represents the rich factor value of the enrichment degree, and the vertical axis represents the GO Term. Of these, the size of the shape represents the number of differentially expressed proteins mapped. The larger the shape, the greater the number. The color of the shape represents the size of the corrected p-value; the redder the color, the smaller the p-value. BP, Biological Process; CC, Cellular Component; MF, Molecular Function. Diagram of the mTOR-signaling pathway in the KEGG database; red boxes indicate DEGs. **(C)** Histogram of KEGG-enrichment analysis of the top 10 target protein pathways affected by Pso. In the figure, the abscissa is −log10 (p-value) and the ordinate is the pathway name. The length of the column represents the size of the p-value; the longer the column, the smaller the p-value; and the darker the color, the more target proteins mapped to this pathway. **(D)** KEGG pathway analysis of metabolic mTOR diagram detailing target proteins; red denotes the target-expressed proteins.

To elucidate the mechanism underlying Pso-induced osteogenic differentiation of PDLSCs, we initially conducted a network pharmacology analysis to predict potential targets of Pso. After deduplication through database queries, we obtained 24 active compound targets and 1,800 chronic periodontitis-related therapeutic targets (Figure 2A). Eight genes were ultimately identified that served as both target genes of active ingredients and genes associated with chronic periodontitis. Enrichment analysis of the target proteins revealed that Pso targeted a variety of immune- and inflammation-related signaling pathways, including the mTOR signaling pathway, Toll-like receptor signaling pathway, and MAPK signaling pathway (Figures 2B–D); and of these, the mTOR signaling pathway was specifically related to osteogenesis (Laplante and Sabatini, 2012).

#### 3.2.2 Use of transcriptomic sequencing to screen pso targets

**FIGURE 3:**
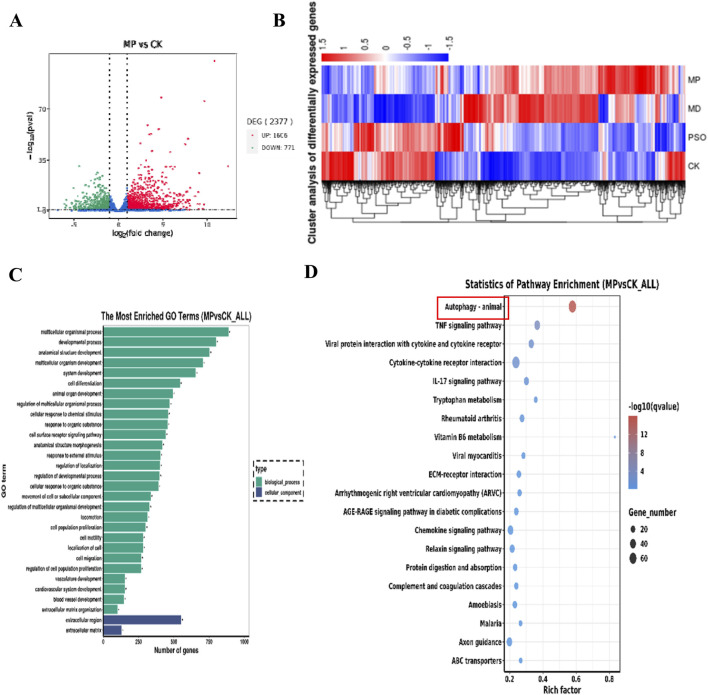
Transcriptomic-sequencing analysis as applied to investigate the effects of Pso on LPS-induced PDLSCs. **(A)** Volcano plot of the number of DEGs; red dots signify upregulated genes, and green dots denote downregulated genes with significant differential expression. **(B)** Differential expression-clustering diagram; the color-gradient transitions from red to blue indicate a diminution in log(FPKM+1) values. **(C)** Top 10 GO functional-enrichment analysis. Green denotes biological processes, while blue signifies cellular components. **(D)** KEGG functional-enrichment analysis features the pathway names on the vertical axis and the rich factor on the horizontal axis; the size of each point reflects the number of DEGs within the corresponding pathway, and the color of the points corresponds to different Q-value ranges. The top 20 significantly enriched pathways were sorted from smallest to largest by Q-value, and the red box in the figure denotes the most obvious pathway for enrichment for visual emphasis, and the red box in the figure denotes the most obvious pathway for enrichment for visual emphasis.

In order to explore the mechanism(s) underlying Pso influences on the osteogenic differentiation of PDLSCs, we sequenced RNA extracted from the cultured cells using an Illumina second-generation high-throughput sequencing platform. This approach allowed us to assess the impact of Pso on PDLSCs, particularly in the context of LPS-induced differential gene expression. In comparison to the control group, we identified 2,377 DEGs in the cells cultured with both LPS/PSO, of which 1,606 were upregulated and 771 were downregulated (all *P* < 0.05) (Figure 3A). When Pso was administered to LPS-induced PDSLCs, the DEGs were predominantly enriched in biological processes, including developmental processes, cell differentiation, and cell regulation (Figure 3B).

In order to further identify the targeting pathway for Pso, we performed GO and KEGG functional-enrichment analyses of the DEGs, and noted that the most significantly enriched differences were observed in the pathways related to autophagy and mitophagy (Figure 3D). Among these factors, the enriched difference in the mTOR pathway was particularly significant, implying that this pathway was critical to the osteogenic process with respect to inflammatory PDLSCs. We therefore speculate that mTOR signaling may constitute a key pathway involved in the osteogenic differentiation of PDLSCs.

#### 3.2.3 Effect of pso on PDLSCs

**FIGURE 4:**
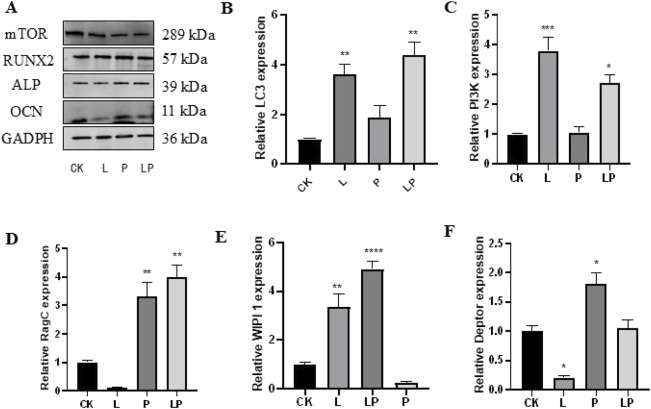
Verification of the effect of Pso on PDLSCs. **(A)** Representative protein expression bands for mTOR, OCN, RUNX2, ALP, and GADPH. **(B–F)**: Expression of LC3, PI3K, Rag C, WIPI 1, and Deptor genes. CK, blank control; L, LPS group; P, Pso group; LP, Pso/LPS co-culture group. Data are presented as mean ± SD. *P < 0.05, **P < 0.01, ***P < 0.001, and ****P < 0.0001 vs. control group.

When we evaluated the expression of related proteins by Western blot analysis, we observed that the expression levels of the osteogenesis-related genes OCN, RUNX2, and ALP were significantly downregulated in the LPS group, while they were elevated following the addition of Pso. Relative to the control group, mTOR expression was notably downregulated in the PSO group. These results suggested that Pso promoted the osteogenic differentiation of PDLSCs, a process that may be associated with mTOR (Figure 4A).

#### 3.2.4 Evaluation of mTOR pathway-related genes

In order to further clarify the mechanism by which mTOR regulated osteogenesis, we evaluated the differential expression of genes related to the mTOR pathway based on the results of transcriptomic sequencing. As can be viewed from the figure, after adding LPS and compared to the control group, the expression levels of LC3 and PI3K increased, while the expression of Deptor diminished; and again compared to the control group, the genes with augmented expression included Deptor, Rag C, and WIPI 1 in the Pso group (Figures 4B–F). These findings were thus consistent with our sequencing results.

### 3.3 Effects of mTOR on osteogenic differentiation in PDLSCs

#### 3.3.1 Effects of an mTOR inhibitor on PDLSCs

**FIGURE 5:**
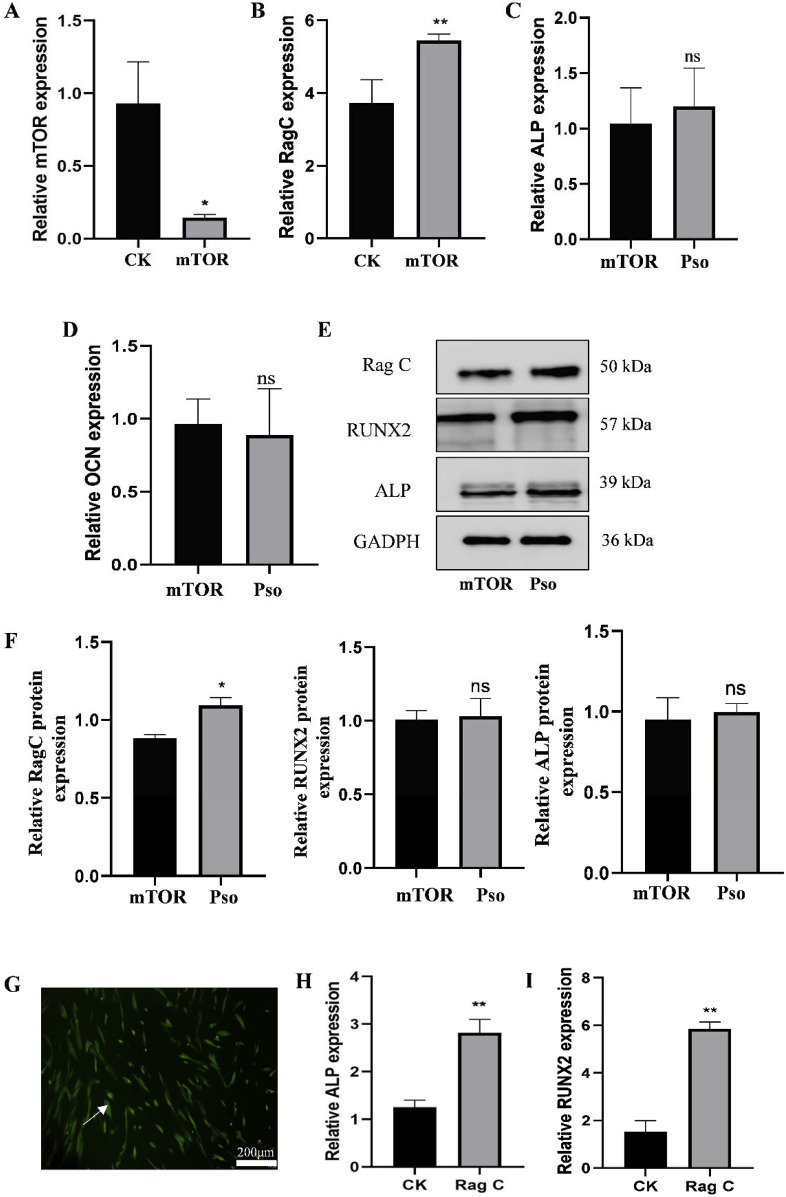
Expression of related genes and proteins after the addition of mTOR inhibitor. **(A)** Expression level of the mTOR gene after adding the inhibitor rapamycin. **(B)** Change in the expression levels of the Rag C gene. **(C–D)** Changes in the expression levels of the osteogenesis-related genes ALP and RUNX2. **(E)** Protein expression levels for of Rag C, Deptor, ALP, and RUNX2. CK, control group; mTOR, mTOR-inhibitor group. **(F)** Relative expression levels of Rag C, ALP, and RUNX2 proteins. mTOR, mTOR-inhibitor group; Pso, mTOR inhibitor + Pso group. **(G)** Representative images of PDLSCs with Rag C lentiviral knockdown; green fluorescence indicates the lentiviral vector particles. **(H–I)** Expression changes in ALP and RUNX2 in PDLSCs with Rag C knockdown. CK, control group; Rag C, PDLSCs (scale bar = 200 μm). Data are presented as mean ± SD. *P < 0.05, **P < 0.01, ***P < 0.001, and ****P < 0.0001 vs. the control group.

In order to examine the relationship between the mTOR-signaling pathway and osteogenesis of PDLSCs, we added rapamycin as an mTOR inhibitor to cultured PDLSCs. Results indicated that the addition of the mTOR inhibitor significantly attenuated the gene-expression level of mTOR (Figure 5A) while simultaneously upregulating Rag C (Figure 5B). We also ascertained that after adding Pso to PDLSCs incubated with the mTOR inhibitor, the expression of Rag C protein was upregulated; and that the gene- and protein-expression levels for the osteogenic markers ALP and RUNX2 tended to increase, but not to a significant degree (Figures 5C–F). This indicated that Pso regulated osteogenic differentiation via mTOR.

#### 3.3.2 Rag C knockdown by lentiviral transfection of PDLSCs

In order to further assess the underlying mechanism of action for Pso in the Rag C/mTOR-signaling axis and to investigate the role of mTOR in the osteogenic differentiation of PDLSCs, we constructed lentiviral vectors to establish PDLSCs with Rag C knockdown. The presence of green fluorescence in the cells validated the success of the lentiviral transfection (Figure 5G), and we observed that the osteogenic genes ALP and RUNX 2 were upregulated after lentiviral knockdown compared to the controls (Figures 5H,I). This result confirmed that Rag C functioned as an upstream regulator of mTOR and that Pso limited mTOR signaling via Rag C, thus modulating the osteogenic differentiation of PDLSCs.

### 3.4 Preparation and application of MPDA-Pso nanoparticles

#### 3.4.1 Preparation and characterization of MPDA-Pso nanoparticles

**FIGURE 6:**
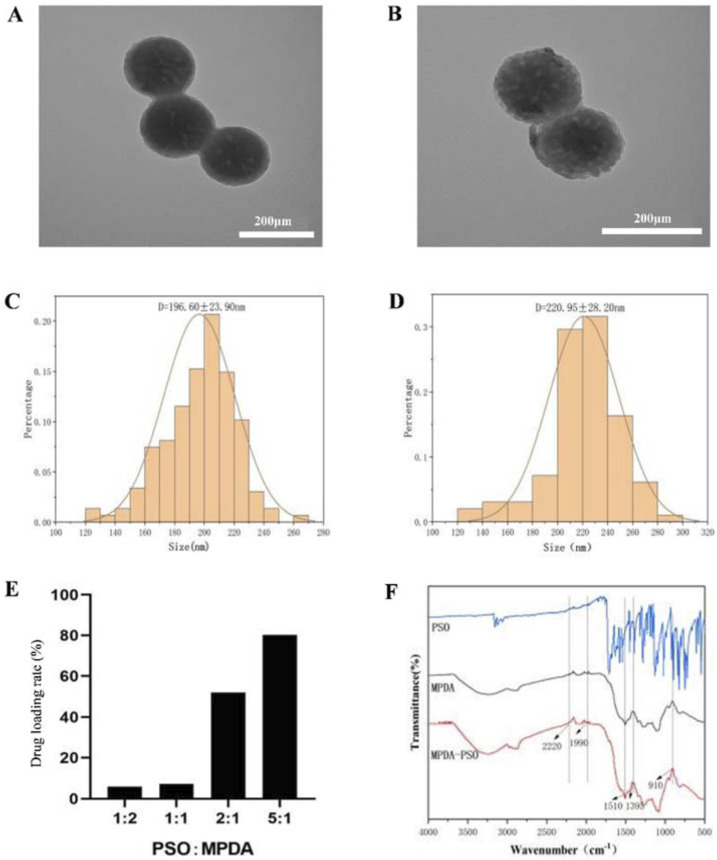
Characterization of MPDA-Pso nanoparticles. **(A)** Representative TEM image of MPDA nanoparticles. **(B)** Representative TEM image of MPDA-Pso nanoparticles. **(C)** Diagram of particle size distribution of MPDA. **(D)** Diagram of particle size distribution of MPDA-Pso nanoparticles. **(E)** Changes in the drug-loading rate of MPDA-Pso nanoparticles prepared with different ratios of Pso/MPDA. **(F)** Images of Fourier transform infrared spectrophotometry (FTIS); the three lines from top to bottom are the absorption peaks of Pso, MPDA, and MPDA-Pso.

In order to improve the physical properties of Pso, we prepared MPDA nanoparticles loaded with Pso. The optimal drug loading rate was approximately 80.30% when the Pso:MPDA ratio was 5:1 (Figure 6E). Under electron microscopy, the prepared MPDA nanoparticles were spherical and showed a pore structure (Figures 6A,C). The mean size of the MPDA nanoparticles was 196.60 ± 21.90 nm, and the zeta potential was −23 mv. Pso was encapsulated in MPDA mesopores, and the MPDA-Pso nanoparticles were spherical in shape (Figure 6B) and of various particle sizes with a mean ± SD of 220.95 ± 28.20 nm (Figure 6D). After drug loading, the particle size and shape did not change significantly, and the zeta potential was −22.7 mv.

Fourier infrared spectra (Figure 6F) showed a series of changes in the MPDA-Pso nanoparticles compared to MPDA alone. The characteristic peak change at 1395 cm-1 represented the bending and tensile vibration of the C-O-H bond, the peak change at 910 cm-1 represented the vibration of the C-O bond of the oxygen-containing functional group in MPDA, and the peak change at 1510 cm-1 represented the shear vibration of the N-H bond. In conclusion, these characteristic peak changes further proved that Pso was successfully loaded and that the MPDA-Pso nanomaterials were prepared successfully.

#### 3.4.2 Effect of MPDA-Pso nanoparticles on osteogenic differentiation of PDLSCs

**FIGURE 7:**
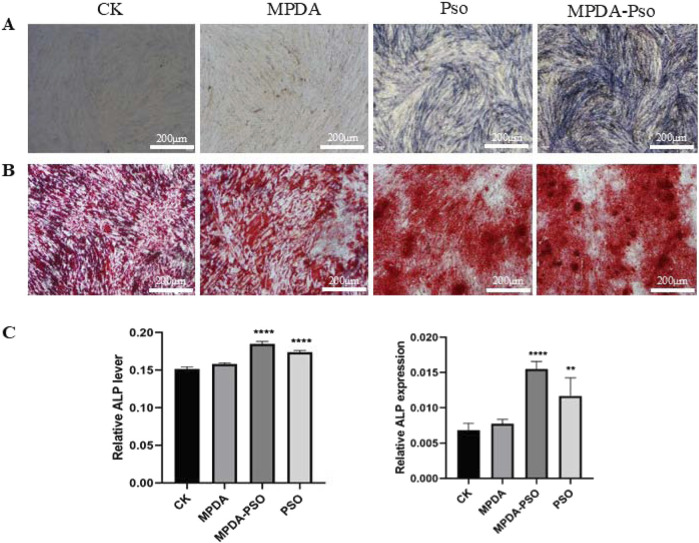
MPDA-Pso promotes osteogenesis of PDLSCs. **(A)** Images of ALP staining. **(B)** Alizarin red-stained images. **(C)** ALP activity and gene-expression analyses (scale bar = 200 μm). Data are presented as mean ± SD. *P < 0.05, **P < 0.01, ***P < 0.001, and ****P < 0.0001 vs. control group.

To verify an effect of the prepared MPDA-Pso nanomaterials on osteogenic differentiation of PDLSCs, MPDA-Pso nanoparticles were co-cultured with PDLSCs, with the control group and the MPDA group displaying no significant blue coloration. In contrast, the cells in both the Pso and MPDA-Pso groups were stained intensely bluish; however, the staining area of the Pso group was notably smaller than that of the MPDA-Pso group (Figure 7A). The results of alizarin red staining mirrored those of ALP staining, with the most prominent red calcium nodules observed in the MPDA-Pso group, while such nodules were infrequently observed in the control and MPDA groups (Figure 7B). After 14 days of cell culture, ALP activity and ALP gene expression were assessed, and we noted significant differences among the Pso group, the MPDA-Pso group, and the control group. Both the Pso group and the MPDA-Pso group exhibited significantly enhanced ALP activity and ALP gene expression in the cells (Figures 7C,D). These results indicated that MPDA-Pso nanomaterials and Pso promoted the osteogenic differentiation of PDLCs.

#### 3.4.3 Effects of MPDA-Pso nanoparticles on osteogenesis in rats with alveolar bone defects

**FIGURE 8:**
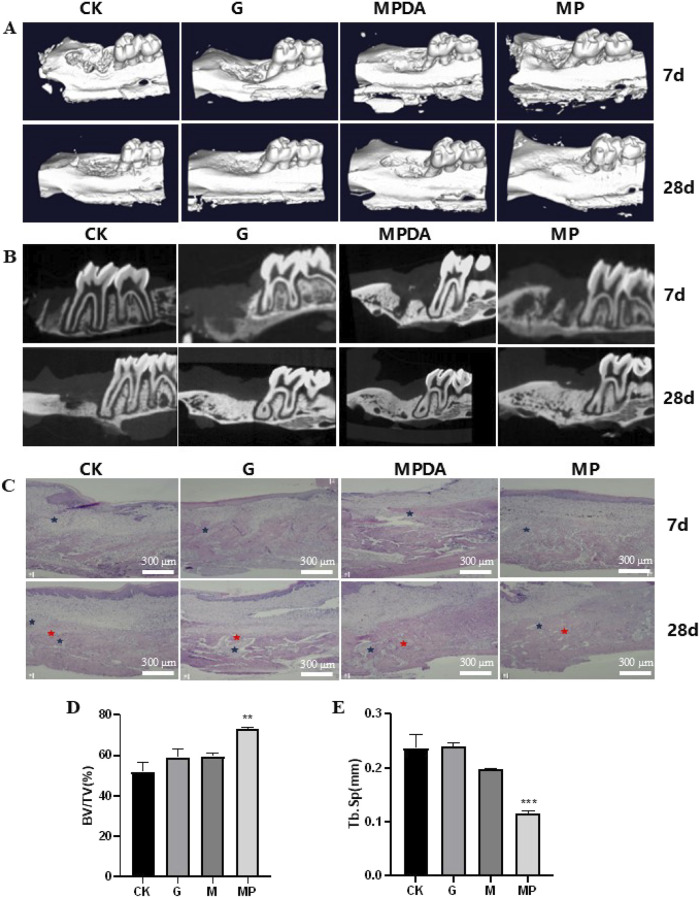
Effect of MPDA-Pso on osteogenesis in defective alveolar bone in rats. **(A–B)**: micro-CT images on days 7 and 28. **(C)**: H&E-stained images of alveolar bones; blue marks indicate inflammatory cell infiltration, and red marks indicate new bone tissue. **(D)**: Trabecular bone parameter BV/TV as obtained by micro-CT analysis. **(E)** Tb. Sp data analysis as obtained by micro-CT analysis. CK, control group; G, gelatin sponge; MPDA, gelatin sponge combined with MPDA; MP, gelatin sponge with MPDA-Pso BV/TV, bone volume per tissue volume; Tb. Sp, trabecular separation (scale bar = 300 μm). Data are expressed as mean ± SD. **P < 0.01 and ***P < 0.001.

To evaluate the osteogenic effects of MPDA-Pso nanomaterials *in vivo*, we constructed a rat model of maxillary first molar defect, conducted micro-CT analysis 7 days and 28 days after the application of the materials, and noted that the amount of new bone in the MPDA-Pso group increased compared to the control group. Alveolar bone healing was also better at 28 days than at 7 days (Figures 8A,B).

To assess the extent of alveolar bone healing, we conducted histologic analyses to evaluate alveolar inflammation and cellular proliferation at seven and 28 days post-reaction, and observed that all groups manifested an inflammatory response at 7 days. Although the proportion of new bone within the alveolar socket had increased significantly in the MPDA-Pso group by 28 days, the remaining three groups demonstrated more pronounced inflammatory responses in alveolar bone compared to the MPDA-Pso group (Figure 8C). We attribute the diminished inflammatory response observed in the MPDA-Pso group to the material’s beneficial effect on the osteogenic differentiation of BMSCs, as it facilitated the transformation of granulation tissue into woven bone.

Our results also revealed that the bone volume fraction (BV/TV) in the MPDA-Pso group was significantly higher than that in the control group (*P* < 0.01) (Figure 8D). However, there was no significant difference in BV/TV between the gelfoam group or the MPDA group compared to the control group. Additionally, trabecular separation (Tb.Sp) in the MPDA-Pso group was significantly lower than in the control group (*P* < 0.001) (Figure 8E). These findings suggested that MPDA-Pso nanomaterials effectively promoted osteogenesis in rat alveolar sockets.

## 4 Discussion

Chronic periodontitis is at a high incidence in the human population, and can cause non-renewable inflammatory destruction of periodontal bone tissue (Van Der Velden, 2000). Additionally, LPS, the primary antigenic component of the outer membrane of Gram-negative bacteria such as Porphyromonas gingivalis, is able to stimulate local periodontal tissue cells and immune cells to produce a large number of inflammatory mediators, thus playing an important role in the pathogenesis of periodontitis (Gasmi et al., 2022; Xu et al., 2020; Zhang et al., 2023). PDLSCs are the principal source of newly attached cells after periodontitis treatment, manifest a variety of differentiative potentials, and can be employed as ideal model cells for regenerative engineering of periodontal tissue (Tomokiyo et al., 2019; Li et al., 2014).

Pso has been confirmed to exert anti-inflammatory, antibacterial, and osteogenic differentiative activities; and is widely applied to diseases such as psoriasis and vitiligo (Olafsson et al., 2023; Thakur et al., 2020). Our previous study also confirmed that systemic application of Pso significantly reduced alveolar bone loss in rats with periodontitis via modulation of the intestinal flora. Based upon the above considerations, we conducted a network pharmacology analysis to further explore the mechanism(s) by which Pso influences the osteogenic differentiation of PDLSCs. Network pharmacology results showed that Pso affected various biologic pathways and processes used by PDLSCs, including autophagy as related to osteogenesis. We therefore hypothesized that the effect of Pso on LPS-induced PDLSCs was associated with autophagy.

Autophagy is a process of cellular self-degradation and recycling of intracellular components, and plays an important role in the pathogenesis of immune regulation, infection, inflammation, tumors, and neurodegenerative and other diseases (Glick et al., 2010; Mizushima and Komatsu, 2011). It is currently postulated that autophagy is a defense-and-stress regulatory mechanism that is bidirectional (Onorati et al., 2018). Some studies have shown that *P*. gingivalis induces the production of autophagosomes (Lee et al., 2018), and there is also evidence that an enhanced autophagic response inhibits the intracellular survival of A. actinomycetes in a patient’s THP-1 monocyte line infected with this bacterial species (Chung et al., 2018). In order to explore the relationships among Pso, autophagy, and periodontitis in an inflammatory environment, we simulated the inflammatory microenvironment characteristic of patients with periodontitis and performed transcriptomic sequencing. Through enrichment analysis and molecular function annotation of the differential genes involved, we discovered that the autophagic process in PDLCs was significantly affected by Pso, and that the mTOR-signaling pathway was crucial to the process. The mTOR-signaling pathway is an important cell-signaling pathway that participates in a variety of biologic processes such as cell growth, autophagy, and metabolism (Liu and Sabatini, 2020; Wang and Zhang, 2019; Kim and mtor, 2015); Yang G et al. found that MTORC1 is not only an executor of RagC phosphorylation, but also achieves feedback regulation of its own activity. This feedback regulation mechanism enables the mTORC1 signaling pathway to more accurately respond to changes in the intracellular and extracellular environment, maintaining the balance of cell growth, proliferation, and metabolism (Yang et al., 2019). and Abudu et al. ascertained that mTORG1 (a WD domain repeat protein of the mTOR family) inhibited mTOR signaling via Rag GTPases (Rags A–D), thereby promoting infrastructural autophagy (Abudu et al., 2024). The NLRP3 inflammasome produced by periodontitis can promote the maturation and secretion of inflammatory cytokines such as IL-1β and IL-18, while autophagosomes can recognize and encapsulate the NLRP3 inflammasome to degrade it, thereby inhibiting the large-scale production of inflammatory cytokines such as IL-1β and IL-18 from the source, effectively reducing inflammatory damage to periodontal tissue (Hashim et al., 2024; Isola et al., 2022). Recent evidence has shown that autophagy is involved in osteogenesis and bone development, and that AMP-activated protein kinase (AMPK) and mTOR are essential for autophagy (Cheng et al., 2019). Huang et al. also discerned that mTOR inactivation inhibited osteoblast proliferation and promoted differentiation (Huang et al., 2015).

To investigate the role of the mTOR-signaling pathway in the osteogenic differentiation of PDLSCs induced by Pso, we added the mTOR-inhibitor rapamycin to the cells. Our results demonstrated that after adding Pso to the PDLSCs and successfully inhibiting mTOR, the gene- and protein-expression levels of the osteogenesis-related factors ALP and RUNX2 tended to be upregulated (although they were not statistically different). In contradistinction, the expression levels of the Rag C gene increased significantly, indicating that Rag C was an upstream regulator of mTOR, and that the mTOR-signaling pathway was key to enhancing the differentiative capability of PDLSCs toward osteogenesis by Pso. This result also provides evidence for the application of Pso in the treatment of periodontitis.

In order to elucidate the upstream and downstream mechanisms underlying Pso effects on the mTOR pathway, we established a lentiviral Rag C-knockdown model using PDLSCs; and our analysis revealed that the expression of the osteogenesis-related genes ALP and RUNX2 was upregulated, indicating the impact of Rag C on mTOR signaling. We therefore speculate that Pso limited mTOR signaling by inhibiting Rag C, and that it modulated autophagy and stem cell osteogenesis by regulating the Rag C/mTOR-signaling axis.

Pso is slightly soluble in water, engendering a challenge to its use as an oral medication. To modify its physical properties so as to improve therapeutic efficacy, we selected MPDA as a carrier and prepared MPDA-Pso nanoparticles. This material exhibited a large surface area, facilitated surface modification, possessed favorable biocompatibility, and was convenient for loading drugs—making it widely available for use as a drug-delivery system (Liu et al., 2021). We synthesized MPDA-Pso nanoparticles that significantly increased the water solubility of Pso, and co-cultured these nanoparticles with PDLSCs. Our results indicated that MPDA-Pso nanoparticles promoted the osteogenic differentiation of PDLSCs, suggesting that, while MPDA-Pso altered the physical properties of Pso, it did not affect the material’s capacity to promote osteogenesis.

We subsequently performed micro-CT scan analysis on the maxillary bones of rats that were raised for 7 and 28 days following the application of the materials. The analysis revealed that alveolar bone healing encompassed several processes, including inflammatory cell infiltration, angiogenesis, fibroblast migration, collagen deposition, and bone remodeling (Thoma et al., 2017), of which the bone-remodeling process was the most significant. These results demonstrate that MPDA-Pso significantly increased the volume of bone mass and significantly reduced trabecular bone separation, demonstrating its potential for effectively promoting alveolar bone formation in rats.

## 5 Conclusion

In summary, we herein demonstrated that Pso promotes the osteogenic differentiation of PDLSCs. Specifically, Pso’s promotional effect on the osteogenic differentiation of inflammatory PDLSCs was generated via its regulation of the Rag C/mTOR-signaling axis and the autophagic process, thus modulating stem cell osteogenesis. Furthermore, the MPDA-Pso nanoparticles we developed were shown to promote osteogenic differentiation of PDLSCs *in vitro* and to facilitate alveolar bone formation in rats *in vivo*. Collectively, our findings offer novel insights into the mechanisms by which Pso enhances osteogenic differentiation of PDLSCs within an inflammatory microenvironment, and potentially facilitates its application in the treatment of chronic periodontitis. Our experiments also revealed that the mechanism whereby Pso promoted osteogenic differentiation of PDLSCs involved a combination of multiple pathways, highlighting the necessity for the further examination of other potential underlying mechanisms.

## Data Availability

The original contributions presented in the study are included in the article/Supplementary Material, further inquiries can be directed to the corresponding authors.

## References

[B1] Abudu Y. P. Kournoutis A. Brenne H. B. Lamark T. Johansen T. (2024). MORG1 limits mTORC1 signaling by inhibiting rag GTPases. Mol. Cell 84 (3), 552–69.e11. 10.1016/j.molcel.2023.11.023 38103557

[B2] Calabrese E. J. (2021). Human periodontal ligament stem cells and hormesis: enhancing cell renewal and cell differentiation. Pharmacol. Res. 173, 105914. 10.1016/j.phrs.2021.105914 34563662

[B3] Chen F. Xing Y. Wang Z. Zheng X. Zhang J. Cai K. (2016). Nanoscale polydopamine (PDA) meets π-π interactions: an interface-directed coassembly approach for mesoporous nanoparticles. Langmuir 32 (46), 12119–12128. 10.1021/acs.langmuir.6b03294 27933877

[B4] Cheng Y. Huang L. Wang Y. Huo Q. Shao Y. Bao H. (2019). Strontium promotes osteogenic differentiation by activating autophagy via the the AMPK/mTOR signaling pathway in MC3T3-E1 cells. Int. J. Mol. Med. 44 (2), 652–660. 10.3892/ijmm.2019.4216 31173178 PMC6605659

[B5] Chung J. Kim S. Lee H. A. Park M. H. Kim S. Song Y. R. (2018). Trans-cinnamic aldehyde inhibits aggregatibacter actinomycetemcomitans-induced inflammation in THP-1-derived macrophages via autophagy activation. J. Periodontol. 89 (10), 1262–1271. 10.1002/JPER.17-0727 29761921

[B6] Edwards S. R. Wandless T. J. (2007). The rapamycin-binding domain of the protein kinase Mammalian target of rapamycin is a destabilizing domain. J. Biol. Chem. 282 (18), 13395–13401. 10.1074/jbc.M700498200 17350953 PMC3763840

[B7] Gasmi B. A. Kumar Mujawdiya P. Noor S. Gasmi A. (2022). Porphyromonas gingivalis in the development of periodontitis: impact on dysbiosis and inflammation. Arch. Razi Inst. 77 (5), 1539–1551. 10.22092/ARI.2021.356596.1875 37123122 PMC10133641

[B8] Glick D. Barth S. Macleod K. F. (2010). Autophagy: cellular and molecular mechanisms. J. Pathol. 221 (1), 3–12. 10.1002/path.2697 20225336 PMC2990190

[B9] Graziani F. Karapetsa D. Alonso B. Herrera D. (2000). Nonsurgical and surgical treatment of periodontitis: how many options for one disease? Periodontol 75 (1), 152–188. 10.1111/prd.12201 28758300

[B10] Guo Y. F. Su T. Yang M. Li C. J. Guo Q. Xiao Y. (2021). The role of autophagy in bone homeostasis. J. Cell Physiol. 236 (6), 4152–4173. 10.1002/jcp.30111 33452680

[B11] Hashim N. Babiker R. Mohammed R. Rehman M. M. Chaitanya N. C. Gobara B. (2024). NLRP3 inflammasome in autoinflammatory diseases and periodontitis advance in the management. J. Pharm. Bioallied Sci. 16 (Suppl. 2), S1110–S1119. 10.4103/jpbs.jpbs_1118_23 38882867 PMC11174327

[B12] Herrera D. Sanz M. Kebschull M. Jepsen S. Sculean A. Berglundh T. (2022). Treatment of stage IV periodontitis: the EFP S3 level clinical practice guideline. J. Clin. Periodontol. 49 (Suppl. 24), 4–71. 10.1111/jcpe.13639 35688447

[B13] Huang B. Wang Y. Wang W. Chen J. Lai P. Liu Z. (2015). mTORC1 prevents preosteoblast differentiation through the notch signaling pathway. PLoS Genet. 11 (8), e1005426. 10.1371/journal.pgen.1005426 26241748 PMC4524707

[B14] Huang Y. Liao L. Su H. Chen X. Jiang T. Liu J. (2021). Psoralen accelerates osteogenic differentiation of human bone marrow mesenchymal stem cells by activating the TGF-β/Smad3 pathway. Exp. Ther. Med. 22 (3), 940. 10.3892/etm.2021.10372 34306204 PMC8281312

[B15] Hu J. Ding Y. Tao B. Yuan Z. Yang Y. Xu K. (2022). Surface modification of titanium substrate via combining photothermal therapy and quorum-sensing-inhibition strategy for improving osseointegration and treating biofilm-associated bacterial infection. Bioact. Mater 18, 228–241. 10.1016/j.bioactmat.2022.03.011 35387171 PMC8961458

[B16] Isola G. Polizzi A. Santonocito S. Alibrandi A. Williams R. C. (2022). Periodontitis activates the NLRP3 inflammasome in serum and saliva. J. Periodontol. 93 (1), 135–145. 10.1002/JPER.21-0049 34008185

[B17] Jamalis J. Yusof F. S. M. Chander S. Wahab R. A. P Bhagwat D. Sankaranarayanan M. (2020). Psoralen derivatives: recent advances of synthetic strategy and pharmacological properties. Antiinflamm. Antiallergy Agents Med. Chem. 19 (3), 222–239. 10.2174/1871523018666190625170802 31241020 PMC7499361

[B18] Jiang S. Zhu F. Ji X. Li J. Tian H. Wang B. (2022). Mesoporous polydopamine-based nanovehicles as a versatile drug loading platform to enable tumor-sufficient synergistic therapy. ChemMedChem 17 (19), e202200360. 10.1002/cmdc.202200360 36000799

[B19] Kim Y. C. mTOR G. K. L. (2015). A pharmacologic target for autophagy regulation. J. Clin. Invest 125 (1), 25–32. 10.1172/JCI73939 25654547 PMC4382265

[B20] Kinane D. F. (2000). Causation and pathogenesis of periodontal disease. Periodontol 2001 (25), 8–20. 10.1034/j.1600-0757.2001.22250102.x 11155179

[B21] Kwon T. Lamster I. B. Levin L. (2021). Current concepts in the management of periodontitis. Int. Dent. J. 71 (6), 462–476. 10.1111/idj.12630 34839889 PMC9275292

[B22] Laplante M. Sabatini D. M. (2012). mTOR signaling in growth control and disease. Cell 149 (2), 274–293. 10.1016/j.cell.2012.03.017 22500797 PMC3331679

[B23] Lee K. Roberts J. S. Choi C. H. Atanasova K. R. Yilmaz Ö. (2018). Porphyromonas gingivalis traffics into endoplasmic reticulum-rich-autophagosomes for successful survival in human gingival epithelial cells. Virulence 9 (1), 845–859. 10.1080/21505594.2018.1454171 29616874 PMC5955440

[B24] Liccardo D. Cannavo A. Spagnuolo G. Ferrara N. Cittadini A. Rengo C. (2019). Periodontal disease: a risk factor for diabetes and cardiovascular disease. Int. J. Mol. Sci. 20 (6), 1414. 10.3390/ijms20061414 30897827 PMC6470716

[B25] Liu G. Y. Jiang X. X. Zhu X. He W. y. Kuang Y. l. Ren K. (2015). ROS activates JNK-mediated autophagy to counteract apoptosis in mouse mesenchymal stem cells *in vitro* . Acta Pharmacol. Sin. 36 (12), 1473–1479. 10.1038/aps.2015.101 26592514 PMC4816227

[B26] Liu G. Y. Sabatini D. M. (2020). mTOR at the nexus of nutrition, growth, ageing and disease. Nat. Rev. Mol. Cell Biol. 21 (4), 183–203. 10.1038/s41580-019-0199-y 31937935 PMC7102936

[B27] Liu H. Xu Y. Cui Q. Liu N. Chu F. Cong B. (2021). Effect of psoralen on the intestinal barrier and alveolar bone loss in rats with chronic periodontitis. Inflammation 44 (5), 1843–1855. 10.1007/s10753-021-01462-7 33839980

[B28] Li X. Yu C. Hu Y. Xia X. Liao Y. Zhang J. (2018). New application of psoralen and angelicin on periodontitis with anti-bacterial, anti-inflammatory, and osteogenesis effects. Front. Cell Infect. Microbiol. 8, 178. 10.3389/fcimb.2018.00178 29922598 PMC5996246

[B29] Li Z. Jiang C. M. An S. Cheng Q. Huang Y. F. Wang Y. T. (2014). Immunomodulatory properties of dental tissue-derived mesenchymal stem cells. Oral Dis. 20 (1), 25–34. 10.1111/odi.12086 23463961

[B30] Ma H. Peng J. Zhang J. Pan L. Ouyang J. Li Z. (2022). Frontiers in preparations and promising applications of mesoporous polydopamine for cancer diagnosis and treatment. Pharmaceutics 15 (1), 15. 10.3390/pharmaceutics15010015 36678644 PMC9861962

[B31] Mizushima N. Komatsu M. (2011). Autophagy: renovation of cells and tissues. Cell 147 (4), 728–741. 10.1016/j.cell.2011.10.026 22078875

[B32] Nuschke A. Rodrigues M. Stolz D. B. Chu C. T. Griffith L. Wells A. (2014). Human mesenchymal stem cells/multipotent stromal cells consume accumulated autophagosomes early in differentiation. Stem Cell Res. Ther. 5 (6), 140. 10.1186/scrt530 25523618 PMC4446103

[B33] Olafsson S. Rodriguez E. Lawson A. R. J. Abascal F. Huber A. R. Suembuel M. (2023). Effects of psoriasis and psoralen exposure on the somatic mutation landscape of the skin. Nat. Genet. 55 (11), 1892–1900. 10.1038/s41588-023-01545-1 37884686 PMC10632143

[B34] Onorati A. V. Dyczynski M. Ojha R. Amaravadi R. K. (2018). Targeting autophagy in cancer. Cancer 124 (16), 3307–3318. 10.1002/cncr.31335 29671878 PMC6108917

[B35] Rangaraju S. Verrier J. D. Madorsky I. Nicks J. Dunn W. A. Notterpek L. (2010). Rapamycin activates autophagy and improves myelination in explant cultures from neuropathic mice. J. Neurosci. 30 (34), 11388–11397. 10.1523/JNEUROSCI.1356-10.2010 20739560 PMC3478092

[B36] Sanz I. Alonso B. Carasol M. Herrera D. Sanz M. (2012). Nonsurgical treatment of periodontitis. J. Evid. Based Dent. Pract. 12 (Suppl. l), 76–86. 10.1016/S1532-3382(12)70019-2 23040340

[B37] Sanz M. Herrera D. Kebschull M. Chapple I. Jepsen S. Beglundh T. (2020b). Treatment of stage I-III periodontitis-the EFP S3 level clinical practice guideline. J. Clin. Periodontol. 47 (22), 4–60. 10.1111/jcpe.13290(32383274 PMC7891343

[B38] Sanz M. Marco Del Castillo A. Jepsen S. Gonzalez-Juanatey J. R. D'Aiuto F. Bouchard P. (2020a). Periodontitis and cardiovascular diseases: consensus report. J. Clin. Periodontol. 47 (3), 268–288. 10.1111/jcpe.13189 32011025 PMC7027895

[B39] Stemmler A. Symmank J. Steinmetz J. von Brandenstein K. Hennig C. L. Jacobs C. (2021). GDF15 supports the inflammatory response of PdL fibroblasts stimulated by P. gingivalis LPS and concurrent compression. Int. J. Mol. Sci. 22 (24), 13608. 10.3390/ijms222413608 34948405 PMC8708878

[B40] Teles F. Collman R. G. Mominkhan D. Wang Y. (2022). Viruses, periodontitis, and comorbidities. Periodontol. 2000 89 (1), 190–206. 10.1111/prd.12435 35244970

[B41] Thakur A. Sharma R. Jaswal V. S. Nepovimova E. Chaudhary A. Kuca K. (2020). Psoralen: a biologically important coumarin with emerging applications. Mini Rev. Med. Chem. 20 (18), 1838–1845. 10.2174/1389557520666200429101053 32348216

[B42] Thoma D. S. Naenni N. Benic G. I. Muñoz F. Hämmerle C. H. F. Jung R. E. (2017). Effect of ridge preservation for early implant placement - is there a need to remove the biomaterial? J. Clin. Periodontol. 44 (5), 556–565. 10.1111/jcpe.12709 28207942

[B43] Tomokiyo A. Wada N. Maeda H. (2019). Periodontal ligament stem cells: regenerative potency in periodontium. Stem Cells Dev. 28 (15), 974–985. 10.1089/scd.2019.0031 31215350

[B44] Van Der Velden U. (2000). What exactly distinguishes aggressive from chronic periodontitis: is it mainly a difference in the degree of bacterial invasiveness? Periodontol 75 (1), 24–44. 10.1111/prd.12202 28758297

[B45] Wang J. Zhang Y. Cao J. Wang Y. Anwar N. Zhang Z. (2023). The role of autophagy in bone metabolism and clinical significance. Autophagy 19 (9), 2409–2427. 10.1080/15548627.2023.2186112 36858962 PMC10392742

[B46] Wang Y. Zhang H. (2019). Regulation of autophagy by mTOR signaling pathway. Adv. Exp. Med. Biol. 1206, 67–83. 10.1007/978-981-15-0602-4_3 31776980

[B47] Xu W. Zhou W. Wang H. Liang S. (2020). Roles of Porphyromonas gingivalis and its virulence factors in periodontitis. Adv. Protein Chem. Struct. Biol. 120, 45–84. 10.1016/bs.apcsb.2019.12.001 32085888 PMC8204362

[B48] Yang G. Humphrey S. J. Murashige D. S. Francis D. Wang Q. P. Cooke K. C. (2019). RagC phosphorylation autoregulates mTOR complex 1. EMBO J. 38 (3), e99548. 10.15252/embj.201899548 30552228 PMC6356064

[B49] Yuan X. Bi Y. Yan Z. Pu W. Li Y. Zhou K. (2016). Psoralen and isopsoralen ameliorate sex hormone deficiency-induced osteoporosis in female and Male mice. Biomed. Res. Int. 2016, 6869452. 10.1155/2016/6869452 27239473 PMC4867056

[B50] Yu M. Wang L. Ba P. Li L. Sun L. Duan X. (2017). Osteoblast progenitors enhance osteogenic differentiation of periodontal ligament stem cells. J. Periodontol. 88 (10), e159–e168. 10.1902/jop.2017.170016 28517970

[B51] Zhang Z. Zhang Y. Cai Y. Li D. He J. Feng Z. (2023). NAT10 regulates the LPS-induced inflammatory response via the NOX2-ROS-NF-κB pathway in macrophages. Biochim. Biophys. Acta Mol. Cell Res. 1870 (7), 119521. 10.1016/j.bbamcr.2023.119521 37307924

[B52] Zhu M. Shi Y. Shan Y. Guo J. Song X. Wu Y. (2021). Recent developments in mesoporous polydopamine-derived nanoplatforms for cancer theranostics. J. Nanobiotechnology 19 (1), 387. 10.1186/s12951-021-01131-9 34819084 PMC8613963

