# Supplementary figures and images for: Psoralen-mediated regulation of osteogenic differentiation of periodontal ligament stem cells: involvement of the mTOR pathway
Source: Front Cell Dev Biol. 2025 Jul 11;13:1634945. doi: 10.3389/fcell.2025.1634945 (PMC12289674; doi:10.3389/fcell.2025.1634945)

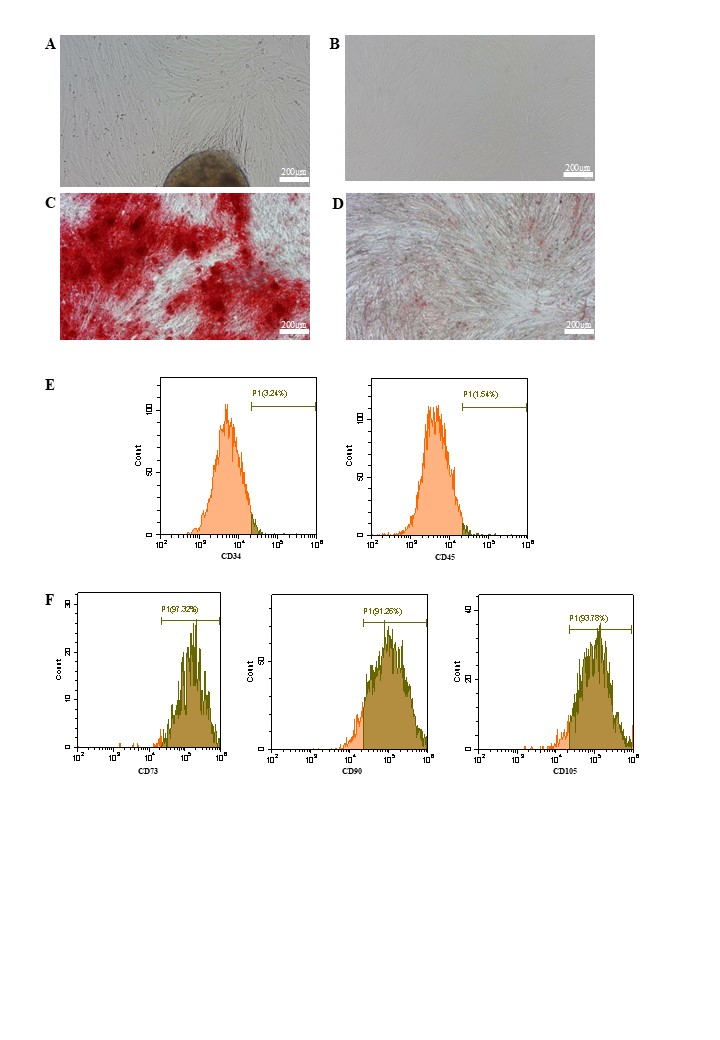

Supplement: Supplementary file 1 [file Image1.jpeg]
